# Microwave Metamaterial Absorber for Non-Destructive Sensing Applications of Grain

**DOI:** 10.3390/s18061912

**Published:** 2018-06-12

**Authors:** Yin Zhang, Junming Zhao, Jie Cao, Bo Mao

**Affiliations:** 1 Collaborative Innovation Center for Modern Grain Circulation and Safety, School of Information Engineering, Nanjing University of Finance and Economics, Nanjing 210046, China; 370820542@163.com (Y.Z.); maoboo@gmail.com (B.M.); 2 Department of Electronic Engineering, School of Electronic Science and Engineering, Nanjing University, Nanjing 210093, China; jmzhao@nju.edu.cn

**Keywords:** metamaterial absorber, resonant frequency, sensing, effective permittivity, grain, non-destructive evaluation

## Abstract

In this work, we propose a metamaterial absorber at microwave frequencies with significant sensitivity and non-destructive sensing capability for grain samples. This absorber is composed of cross-resonators periodically arranged on an ultrathin substrate, a sensing layer filled with grain samples, and a metal ground. The cross-resonator array is fabricated using the printed circuit board process on an FR-4 board. The performance of the proposed metamaterial is demonstrated with both full-wave simulation and measurement results, and the working mechanism is revealed through multi-reflection interference theory. It can serve as a non-contact sensor for food quality control such as adulteration, variety, etc. by detecting shifts in the resonant frequencies. As a direct application, it is shown that the resonant frequency displays a significant blue shift from 7.11 GHz to 7.52 GHz when the mass fraction of stale rice in the mixture of fresh and stale rice is changed from 0% to 100%. In addition, the absorber shows a distinct difference in the resonant absorption frequency for different varieties of grain, which also makes it a candidate for a grain classification sensor. The presented scheme could open up opportunities for microwave metamaterial absorbers to be applied as efficient sensors in the non-destructive evaluation of agricultural and food product quality.

## 1. Introduction

Over the last several decades, metamaterials, which are a new type of synthetic electromagnetic (EM) media with periodic or quasi-periodic structures fabricated or synthesized by artificial methods, have attracted considerable attention due to their fascinating capabilities, which are unavailable from natural materials, and their extraordinary abilities related to manipulating EM waves [1,2,3,4,5,6]. One of the great advantages of metamaterials is that the interaction between EM waves and substances can be successfully tailored by artificially designing or revising unit cells, which provides a great possibility for sensing applications of metamaterials [7,8,9]. In fact, with continuous progress in the design and fabrication of metamaterials, the interdisciplinary area between metamaterials and sensing technology has become a fertile ground for the development of new science and technology [10,11,12,13]. Currently, significant advancements have been made on metamaterial-based sensors, with a level of performance that is able to detect substance information, and interesting and useful sensing applications have been demonstrated for such applications as chemical, food, and biosensing devices [14,15,16,17,18,19,20,21]. Among these sensors, metamaterial absorbers show promising prospects and great application potential in developing high-performance sensing devices [22,23,24].

Metamaterial absorbers primarily depend on their strong resonance characteristics to gain a significant advantage, since this type of sensor can produce a strong and measurable readout signal with a resonance absorption peak that is strong enough to accurately track the parameter shift in reflection spectra [25,26,27]. When the dielectric substrate or geometric structure changes under an external drive, the equivalent electromagnetic parameters of the metamaterial will be altered accordingly, resulting in a variation in the resonance frequency and absorption amplitude. If there is a one-to-one corresponding relation between the variation of frequency or absorption and the external drive, metamaterial absorbers can be used to detect changes in the parameters of the drive, such as the dielectric constant, substrate thickness, biological features, etc. [28,29,30]. However, the research on the sensing applications of metamaterial have mainly focused on the terahertz and infrared bands [31,32,33,34], while works on the microwave waveband are still lagging behind relatively speaking, limiting more widespread applications. Furthermore, most of the microwave and metamaterial absorber-based sensors usually require specific samples pre-treated by destructive methods or liquid samples, due to the narrow sensing area, which also limits their suitability in non-destructive sensing applications for solid food.

Food safety is a significant public health issue that in recent years has been a source of increasing concern for governments, consumers, and the food industry. In particular, the quality control of grain, as the most important component of agricultural food products, cannot be ignored [35]. The traditional analytical methods of grain quality control mainly rely on organic inspection or chemical analysis, which inevitably cause high error or damage to the tested samples [36]. Thus, non-destructive detection techniques are in high demand, and progress in non-destructive food quality evaluation has been made owing to the sustained development of modern analytical methods and instruments, e.g., gas chromatography, mass spectrometry, electronic noses, and electronic tongues [37]. However, these techniques usually require complicated sample pre-treatment and data processing, as well as sophisticated instrumentation [38], which could be time-consuming and are relatively expensive in daily use.

In the present study, we introduce a metamaterial absorber for granulated grain sensor applications with a non-destructive approach in the microwave band. The proposed metamaterial absorber mainly consists of a cross-resonator array (CRA), a grain sample, and a metal plate, with the microstructure fabricated using the printed circuit board (PCB) process. The proposed absorber can be used as a non-contact sensor for grain quality evaluation, including adulteration and variety. As a concrete example, we employ both numerical simulations and experiments to demonstrate that the absorber can quantificationally detect the adulteration of rice (mass fraction of stale rice in the mixture of fresh and stale rice) by measuring changes in the resonant frequencies. At a resonance frequency, there is a significant absorption peak for the incident EM wave energy, so the mass fraction of stale rice can be determined by confirming the absorption frequency. As another example, this absorber can distinguish the varieties of grain according to the differences in the resonant absorption frequency corresponding to different grain samples. We also used multi-reflection interference theory to investigate the physical insights of our design. The proposed metamaterial absorber has the advantages of rapidity, significant sensitivity, and non-destructive sensing capability, and could expand the sensing application range of metamaterial absorbers at microwave frequencies.

## 2. Structure and Methods

### 2.1. Structure Design and Simulation Method

**Figure 1:**
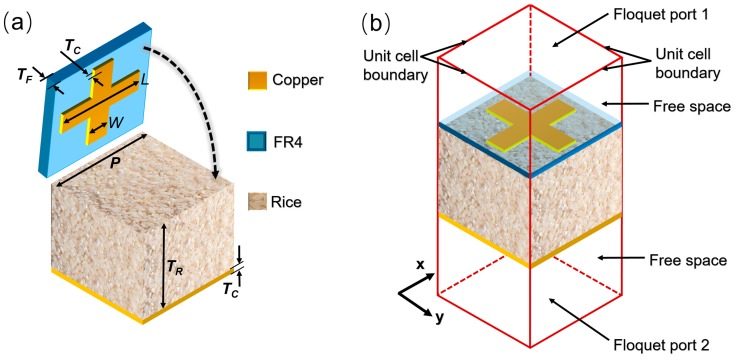
Schematic of (**a**) unit cell; (**b**) simulation setup.

The proposed metamaterial absorber is composed of three layers, namely a CRA with an ultrathin substrate on top, a grain sample as a dielectric layer in the middle, and a metal ground plate at the bottom. In order to clearly show its unit cell design, the top layer is segregated from the grain layer, and schematically shown in Figure 1a. The unit cell of the metamaterial absorber is formed after tightly stacking all of the layers together, as exhibited in Figure 1b. The metallic cross-resonator abutting the grain is designed on an FR-4 dielectric substrate with a permittivity *ε* = 4.4 − 0.02*j* and a thickness *T_F_*= 1 mm. Both the metallic cross-resonator and bottom ground plate are modeled from a Cu sheet with a conductivity of 5.8 × 10^7^ S/m and a thickness *T_c_* = 0.017 mm. The thickness of the Cu sheet used here is much larger than the typical skin depth in the microwave regime, so that the reflection is the only factor limiting the absorption. The geometric parameters of the unit cell are determined after performance optimization through EM simulation. The width *W* and length *L* of the Cu cross-wire are 1 mm and 4.5 mm, respectively. The period *P* of the square unit cell is 5 mm. The thickness *T_R_* of the grain layer is 26 mm. This absorber can sense changes in the effective permittivity for different grain samples by detecting shifts in the resonant frequencies, and the effective permittivity of grain will vary across the difference in grain quality. Thus, it can be used for a non-contact sensor in grain quality control.

In order to prove the performance of the proposed absorber, simulation experiments based on CST MICROWAVE STUDIO^®^ were performed. Figure 1b shows the simulation setup in the software. The unit cell is considered an infinite periodic structure that is applied with unit cell boundary conditions along both the *x* and *y* directions, while remaining open (add space) for the *z* direction in free space. The Floquet port excitations with either *x* or *y* polarization are utilized to be the source or detector of EM wave energy. The distance between the wave source and the structure is 6.5 mm. The frequency domain solver is employed to carry out the specific calculation and correspondingly obtain the required results of the simulation model under the illumination of linearly polarized microwaves propagating along the *z* direction.

### 2.2. Experimental Scheme and Method

**Figure 2:**
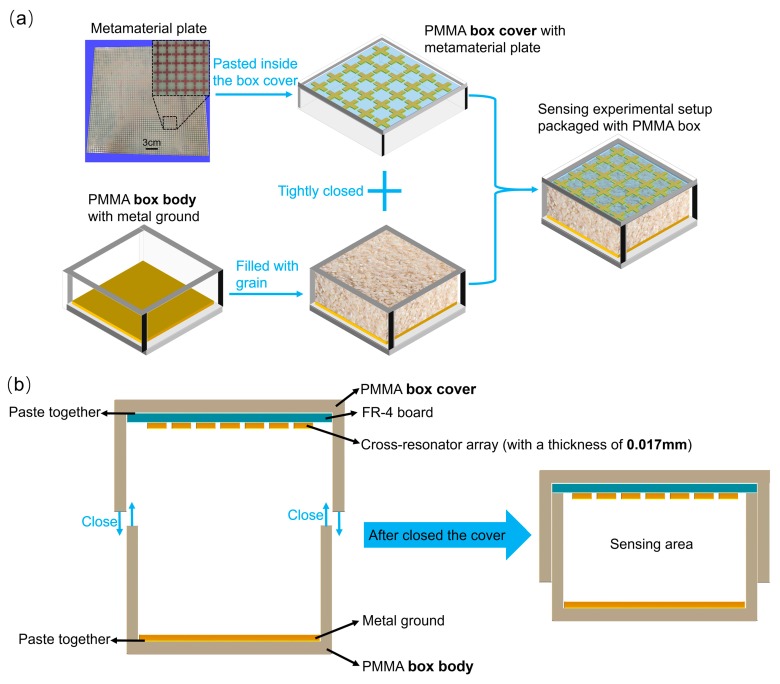
(**a**) The metamaterial plate and its partial enlarged microscope view, as well as the schematic of the packaging process of the sensing experimental setup; (**b**) the method that the top layer of the sensor is connected to the bottom layer of the sensor.

The CRA on the top layer is fabricated by the PCB process on an FR-4 board with a sub-wavelength thickness of approximately 1/41 of the central wavelength, and the corresponding sample plate is shown in Figure 2a. Before starting the experimental measurement, the metamaterial plate, grain sample, and metal ground plate need to be packaged in a polymethyl methacrylate (PMMA) box to form the sensing experimental setup with a uniformly square layered structure, where the area filled with grain serves as the sensing layer. The schematic of the packaging process and sensing experimental setup are shown in Figure 2a. First, a single-sided copper-clad plate (copper layer up) is pasted at the bottom of PMMA box body as a metal ground. Then, the grains are slowly loaded into the box body through a funnel until they completely fill the space of the box body and go beyond the upper edges. A rectangular hard board is used to scrape the redundant grains along a parallel pair of upper edges of the box body, which makes the top surface of the grains flat and flush with the upper edges of the box body. Therefore, the grains are in a naturally filled state without any compaction in the sensing area. At the same time, the metamaterial plate (CRA down) is pasted inside the box cover. Finally, the box body is tightly closed with the box cover to form a metamaterial absorber as the sensing experimental setup, as shown in Figure 2b, which shows more details about the method through which the top layer of the sensor is connected to the bottom layer. Therefore, the volume of grain samples is fixed for all of the tests, and depends on the internal volume of the PMMA packaging box, which is about 300 mm × 300 mm × 24 mm.

**Figure 3:**
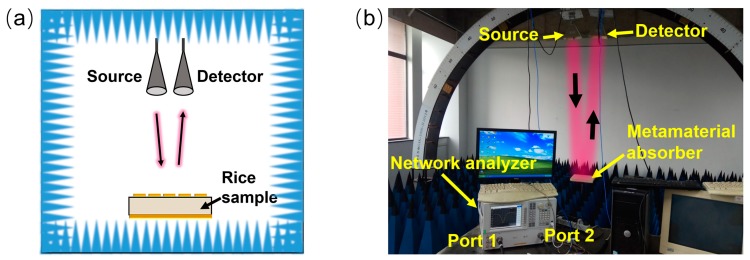
(**a**) Schematic of measurement; (**b**) arch-framing test system.

To investigate the performance of the proposed metamaterial absorber through the experiment, the reflection spectra was measured using the free-space method, a schematic of which is displayed in Figure 3a. The entire packaged structure is placed on a sample stage above a large area of wedge-shaped absorbing materials, which can prevent unwanted reflection signals. Two standard horn antennas are placed about 200 cm away from the wave absorber. The width, depth, and height of the horn antenna are 24.4 cm, 27.9 cm, and 15.9 cm, and the incidence angles of the two horn antennas are ±5°, respectively. The measuring process will be operated in the arch-framing test system that is exhibited in Figure 3b. The test flow is performed according to the system configuration. First, a microwave signal with frequency *f* is generated by the sweep source provided by the vector network analyzer, and fed from port 1 to the source horn antenna via a coaxial transmission line. Then, the linearly polarized plane wave is radiated into the space by the source antenna, and immediately normally impinges on the sample. Finally, the reflected signal enters port 2 of the vector network analyzer via a coaxial transmission line after being received by the detector horn antenna, and the reflection spectra are obtained through the vector network analyzer. Before measuring the sample’s reflection spectra, the empty PMMA box with the metal ground plate at the bottom is utilized to normalize the reflection data.

## 3. Results and Discussions

### 3.1. Non-Destructive Sensing Application for Grain Adulteration

**Figure 4:**
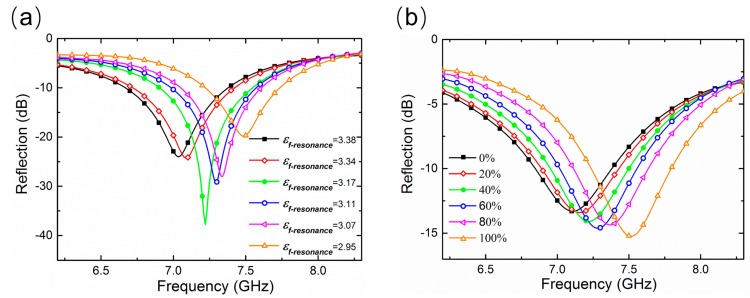
(**a**) Simulated reflection spectra; (**b**) measured reflection spectra for proposed absorber filled with rice samples with different mass fractions of stale rice. It’s worth noting that the rice samples with different mass percentages of stale rice have different effective dielectric constants.

As a proof-of-concept example, we investigated the mixed samples of fresh and stale rice with different mass fractions of stale rice by using the designed absorber to reveal the non-destructive sensing ability of the proposed metamaterial absorber. In the simulation, we first assume that a plane wave normally impinges on the metamaterial absorber. By employing CST MICROWAVE STUDIO^®^ for the full-wave numerical simulations, the reflection spectra for the absorber filled with different rice samples were calculated, and are displayed in Figure 4a. In addition, the measured reflection spectra corresponding to different rice samples were obtained by microwave experiments, and are illustrated in Figure 4b. We observe that the measured reflection spectra demonstrate the resonant frequency increases from 7.11 GHz to 7.52 GHz when the mass fraction of stale rice in the mixture of fresh and stale rice is altered from 0% to 100%, which roughly agree with the simulation results. From Figure 4, we can see that there are some differences between the experiment and simulation results, especially in the resonance amplitude, which is mainly because the simulations are achieved on the approximate equivalent permittivity of grain, whose real and imaginary parts are slightly different from the actual values, resulting in a difference of impedance matching and loss between simulation and experiment. However, the frequency shift range of the experiment and simulation results are basically the same. Furthermore, as shown in the experimental results, there are significant absorption peaks with reflection reductions below −10 dB at different resonant frequencies corresponding to different mass fractions of stale rice, which provide a strong and measurable sensing signal to accurately track the frequency shift in the reflection spectra. The resonant absorption should be polarization independent due to the *C*4 symmetry of the unit cell structure [39].

**Figure 5:**
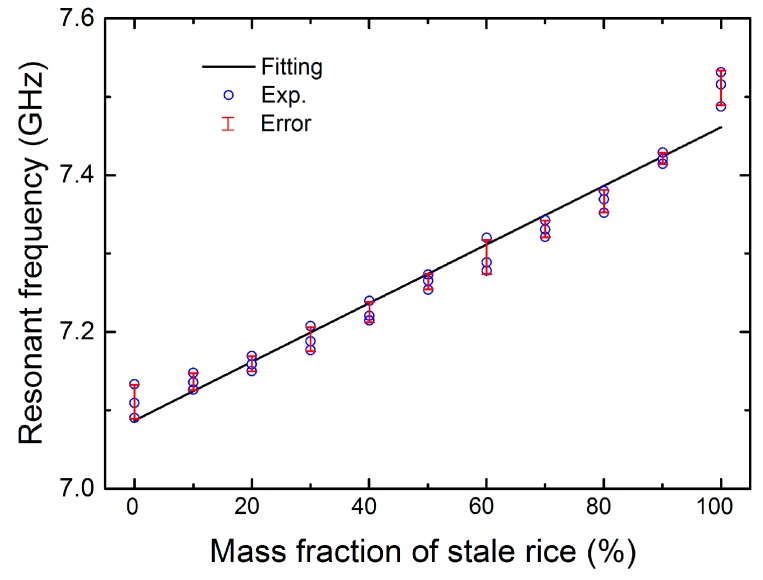
Relationship of the resonance frequency and the mass fraction of stale rice.

To reveal the relationship between the stale-rice percentage and frequency, we plotted the measured resonant frequencies of the presented absorbers for different mass fractions of stale rice in Figure 5, where the black line shows the linear fitting for the experimental data. The fitting function is described by *F =* 0.3742*m* + 7.0869, where the dependent variable *F* is the resonance frequency and the independent variable *m* represents the mass percentage of stale rice. It can be observed from Figure 5 that the experimental data roughly match the linear function. As shown in Figure 5, the experimental results have a relatively large deviation from the fitting curve at mass fractions of 0% and 100%. However, the measured relationship is very similar to the linear function when the mass fraction is altered from 10% to 90%. In addition, in order to investigate the actual minimum resolution of the proposed absorber, we measured the frequency shift for a smaller change of the mass fraction. When the single variation of mass fraction is less than 5%, the frequency shift is minute, and it is difficult to accurately characterize small changes in the mass fraction. Therefore, a 5% mass fraction could be the minimum resolution as the proposed sensor. Overall, by monitoring the resonant frequency from reflection spectra, the mass percentage of stale rice can be identified from a variation of the resonant frequency. This suggests that the proposed metamaterial absorber has potential as a grain sensor application for the non-destructive evaluation of adulteration.

### 3.2. Non-Destructive Sensing Application for Grain Classification

**Figure 6:**
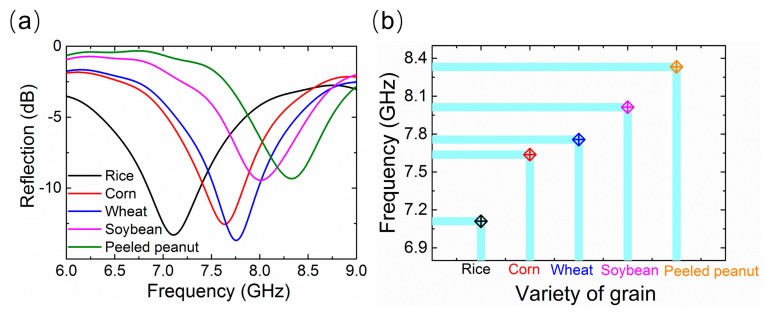
(**a**) Measured reflection spectra for proposed absorber filled with different varieties of grain samples; (**b**) classification results for grains based on the resonance frequency of the metamaterial absorber.

In order to further demonstrate the non-destructive grain sensing applications of the proposed metamaterial absorber, we also explored another direct example of a grain classification sensor application. In this case, the absorber was filled with different varieties of grain samples, including rice, corn, wheat, soybean, and shelled peanuts, and then, the corresponding reflection spectra were separately measured using the arch-framing test system. As shown in Figure 6a, the absorber shows obvious differences in the resonant absorption frequency for different varieties of grain, which makes it a potential candidate for a grain classification sensor. A simple attempt to classify grain was carried out with characteristic resonant frequencies corresponding to different samples, as illustrated in Figure 6b. It was found that satisfactory classification results were presented for the rice, corn, wheat, soybean, and shelled peanut samples. Thus, using resonant frequency signals, rapid and non-destructive grain classification was achieved by employing the proposed metamaterial absorber.

### 3.3. Working Mechanism of Metamaterial Absorber

**Figure 7:**
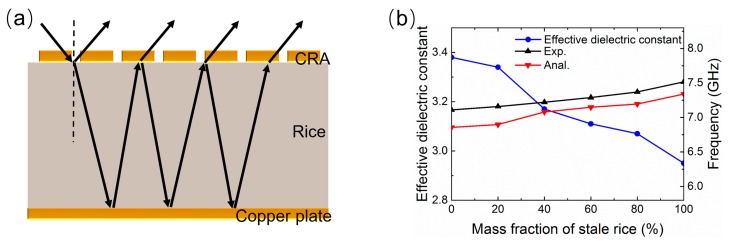
(**a**) Multiple reflections and interference model of Fabry–Pérot-like cavity formed by metamaterial absorber; (**b**) equivalent dielectric constant of different rice samples at the respective resonance absorption frequencies, as well as measured (black) and analyzed resonance absorption frequencies (red) of the absorber as a function of the mass fraction of stale rice.

To elucidate the working mechanism of the proposed metamaterial-based sensor, taking the sensing application for grain adulteration as an example, we considered the entire structure as a cavity model containing two interfaces: the top CRA layer and the bottom copper plate, as displayed in Figure 7a. At the air–rice interface with the CRA, the incident microwave is partially reflected back to air, and partially transmitted into the rice. The transmitted wave continues to propagate until it reaches the copper plate. After the reflection reaches the copper plate and propagates into the rice, partial reflection and transmission occur again at the air–rice interface with the CRA [40]. According to multi-reflection interference theory [40], the EM waves will travel back and forth between the top and bottom interfaces, forming a Fabry–Pérot-like resonator and resulting in multiple backward-reflection beams of the entire structure. The overall reflection is then the superposition of the multiple backward-reflection beams. At resonance frequencies, the destructive interference of the multi-reflection beams leads to nearly zero overall reflection, and is therefore the effect of strongly resonant absorption at these specific frequencies [41]. The resonance absorption frequency can be estimated from the resonance condition of the Fabry–Pérot cavity by the following expression:

(1) $$f=n\frac{c}{2d\sqrt{\varepsilon_{r}}}$$

where *n* is a positive integer, *n* = 2 for the proposed metamaterial absorber, *c* refers to the speed of light in a vacuum, and *d* and *ε_r_* are the thickness and effective dielectric constant of the rice layer, respectively. It can be seen from the above expression that the resonance frequency negatively correlates with the effective dielectric constant of the rice sample. Therefore, decreasing the effective dielectric constant will result in an increase of the resonance frequency. According to the dielectric constant variations of the rice samples with the different mass percentages of stale rice shown in Figure 7b, the effective dielectric constant *ε_r_* will decrease as the mass fraction increases. Here, the dielectric constants of different rice samples are measured through the free-space microwave transmission technique reported by Trabelsi et al. [42] Therefore, increasing the mass percentage of stale rice will increase the resonance frequency, and vice versa. The theoretical analysis and experimental measurement of the resonance frequency are compared in Figure 7b, which demonstrates a similar variation trend, and the discrepancy between them is always less than 5%. Therefore, the adulteration of rice can be identified by detecting shifts in the resonant frequencies.

## 4. Conclusions

In summary, we have proposed a general scheme to design a metamaterial absorber-based sensor for non-destructive sensing applications of grains at microwave frequencies. The physical structure consists of a cross-resonator array, a grain sample, metal ground, and a PMMA box. To demonstrate the sensor’s performance, the top CRA was fabricated on an FR-4 board using the PCB process, and packaged with another two layers in a PMMA box. This metamaterial device can conveniently achieve non-destructive sensing in grain quality control, such as sensing adulteration and variety, by measuring the resonance frequency. As proof-of-concept examples, the absorber was filled with a mixture of fresh and stale rice and investigated through both numerical simulations and experiments. The results show that the resonant frequency of the absorber changed with the variation of effective permittivity for the sensing layer, which is determined by the mass fraction of stale rice in the filling sample. Multi-reflection interference theory and the effective dielectric constant of the rice samples were employed to explain the working mechanism of the proposed metamaterial absorber. Finally, the sensor’s ability to classify different varieties of grain was also demonstrated through experimental measurements. Our sensor design may provide an attractive avenue for applying metamaterial absorbers to the non-destructive sensing of food quality control, and could offer a helpful complement to sensor devices in the microwave regime.
